# Acute rheumatic fever: clinical profile in children in western Ukraine

**Published:** 2017

**Authors:** O Boyarchuk, S Boytsanyuk, T Hariyan

**Affiliations:** * Department of Children’s Diseases and Pediatric Surgery, I. Horbachevsky Ternopil State Medical University, Ternopil, Ukraine

**Keywords:** acute rheumatic fever, children, Jones criteria

## Abstract

Acute rheumatic fever (ARF) may have different clinical manifestations in different countries according to the genetic predisposition, prevalence of rheumatogenic strains, social and economic conditions.

The purpose of this study was to determine the clinical characteristics of ARF in Western Ukraine and to improve the detection of the cases.

A retrospective analysis of 85 medical clinical cases of in-hospital patients aged from 4 to 17 years old was performed. The cases covered patients who underwent treatment in the City Children’s Hospital of Ternopil during 2000 and 2013 with the ARF diagnosis, which was established according to Jones criteria.

65.9% of the ARF patients were admitted to the hospital from October to March. Fever (65.9%) and joint syndrome (78.8%) were the most common causes for admission to the medical care. The admission diagnosis was wrong in 34 (40.0%) children who underwent the treatment. The most frequent major Jones criteria of ARF were carditis (84.7%) and polyarthritis (54.1%). Chorea was significantly less common than carditis (р < 0,001). The adequate treatment of the preceding streptococcal infection was administered in 25 children (53.2%).

Conclusions: The significant incidence of misdiagnoses in the ARF children during admission to the hospital, especially the interpretation of joint syndrome, indicates that physicians need an extra awareness. The lack of specific clinical signs of rheumatic carditis makes it a diagnostic challenge. The revised Jones criteria (2015) for the diagnosis of ARF can improve carditis detection.

The adequate treatment of the preceding streptococcal infection may prevent ARF.

**Abbreviations:** ARF = acute rheumatic fever

## Introduction

Acute rheumatic fever (ARF) was a major public health problem during the last century and a leading cause of cardiovascular morbidity among children and people aged over 40 years [**1**]. The appropriate treatment of streptococcal throat infection, primary and secondary prevention of ARF reduced its occurrence in the recent years [**2**].

Poor socioeconomic status, undernutrition, overcrowded homes, are the most common risk factors among ARF cases [**3**]. Reduced crowding in homes and in schools, better hygiene and increased availability of children’s health care are additional factors contributing to the decreased incidence of this disease [**4**]. A long-term study has also shown the decreased incidence of ARF in children from middle- to high-income families, with access to competent medical care [**5**]. However, ARF remains a major problem among children from the developing countries, especially those with tropical climate [**6**]. The highest documented rates in the world have been found in Maori and Pacific people in New Zealand, in Aboriginal Australians, and in the Pacific Islands nations [**6**,**7**].

WHO experts claim that reliable data for ARF incidence is scarce. There are wide variations between countries, even between population groups in the same country (The World Health Report 2001). ARF has become a rare disease in high-income countries [**8**] as well as in Ukraine. In Ternopil region, which is located in Western Ukraine, a 15-fold reduction of the ARF incidence was observed over the last 10 years – from 0.15 per 1000 children aged below 15 years in 2002, to 0.01 in 2011 [**9**].

ARF may have different clinical manifestations in different countries according to genetic predisposition, prevalence of rheumatogenic strains, social and economic conditions [**8**]. There are also differences in the prevalence of Jones criteria on different continents [**2**], which may be explained by epitopes of rheumatogenic streptococcal strains and genetics [**8**].

Studies of the course and features of this disease are especially important for the early diagnosis. Decreased ARF incidence has led to low alertness of family doctors and pediatricians regarding this disease.

The purpose of this study was to determine the clinical characteristics of ARF in Western Ukraine and to improve the case detection.

## Methods

A retrospective analysis of 85 medical clinical cases of the in-hospital patients aged from 4 to 17 years was performed. The cases covered patients who underwent treatment in the City Children’s Hospital of Ternopil during 2000 and 2013 with the diagnosis of ARF, which was established according to Jones criteria.

All the patients were examined according to the standardized protocol, which consisted of a detailed medical history recorded by a physician, general and special tests, including ECG, echocardiography, Doppler echocardiography (from 2007), anti-streptolysin-O level (ASO), throat culture for Group A beta-hemolytic streptococci.

The scientific ethics committee of Ternopil State Medical University provided the ethical approval for the study. The study conformed to the principles outlined in the declaration of Helsinki.

The data were analyzed by using STATISTICA StatSoft 6.0 software package. The prevalence of variables was assessed by Chi-square test and the Fisher’s exact test. The results were considered significant at α = 0.05 level.

## Results

Baseline characteristics of the patients are listed in **Table 1**.

**Table 1:** Baseline characteristics of patients with ARF

Parameter	ARF (n, % of the patients)
Male	47 (55,3) Female
38 (44,7) Age (years) mean/ range)	10,5 (4-17)

Most children were diagnosed with ARF between the ages of 9 to 11. The mean age of ARF cases was 10.5 ± 1.85 years, 47 being males (55.3 %).

**Fig. 1:**
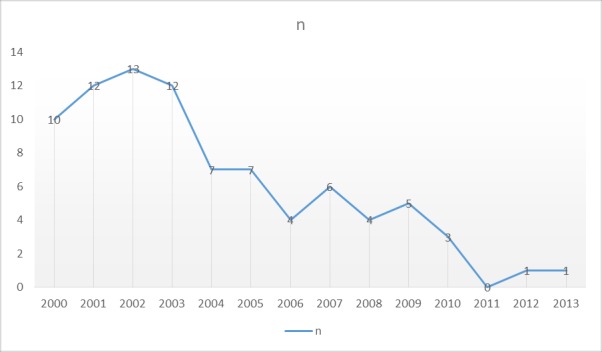
Distribution of ARF patients according to the cases per year (n)

On average, 6.1 patients per year (range: 0 to 13) were diagnosed with ARF (**Fig. 1**). There were 11.75 cases per year within the period of 2000 and 2003, 6.0 cases per year between 2004 and 2007, 4.0 cases per year between 2008 and 2010 and 0.7 cases per year between 2011 and 2013.

**Fig. 2:**
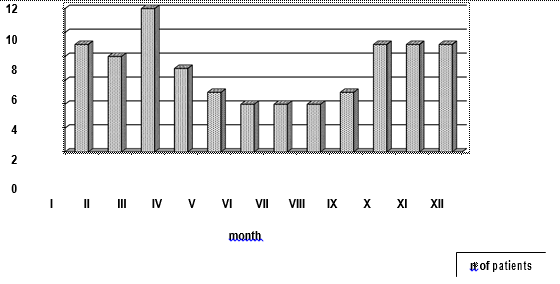
Distribution of ARF patients according to the months of the years (n)

Seasonal differences are shown in **Fig. 2**.

The majority (56/ 65.9%) of the ARF patients were admitted to the hospital from October to March, with the peak admittance in March, while only 29 patients (34.1%) were admitted from April to September (р = 0.02).

Fever (65.9%) and joint syndrome (78.8%) were the most common causes for seeking medical care. The admission diagnosis was wrong in 34 children (40.0%) who underwent the treatment. The most common mistake was the incorrect interpretation of joint syndrome.

The frequency of Jones criteria for ARF diagnosis is shown in **Table 2**. Carditis (84.7%), followed by polyarthritis (54.1%) were the most frequent Jones criteria of ARF. Chorea occurred in significantly fewer cases than carditis (25.9%, р < 0.001). Subcutaneous nodules and erythema marginatum were rarely observed (5.9%, 8.2% respectively). One major criterion was diagnosed in 28.2% of the patients (n = 24): in 17.6% carditis (n = 15) and 10.6% isolated chorea (n = 9). Two major criteria were found in 64.7% of the patients (n = 55): carditis and polyarthritis in 44.7% (n = 38); carditis and chorea in 10.6 % (n = 9). Other combinations occurred more rarely. Three major criteria were found in 7.1% of the patients (n = 6). in Some patients presented different combinations: carditis, polyarthritis, and chorea, while, in other cases, carditis and polyarthritis were combined with erythema marginatum or rheumatic nodules.

**Table 2:** Jones criteria in patients with acute rheumatic fever

Criteria	All patients (n, % of the patients)
Major Jones criteria	
Carditis	72 (84,7)
Polyarthritis	46 (54,1)
Chorea	22 (25,9)
isolated chorea	9 (10,6)
Subcutaneous nodules	5 (5,9) Erythema
marginatum	7 (8,2) Minor Jones criteria
Clinical findings	
Fever	56 (65,9) Arthralgia
Laboratory findings	
Raised ESR	57 (67,1) Raised C-
reactive protein	54 (63,5)
Prolonged PR interval on ECG	10 (11,8)

The complaints of the patients with rheumatic carditis were not specific. On admission to the hospital, only 14 patients (16.5%) complained of chest pain. Intensive, blowing systolic murmur, typical for endocarditis was found in 34 children (40.0%) with signs of carditis. In other children, systolic murmur over the apex and/ or Erb’s point was semi-intense, with irradiation to axilla. Tachycardia and decreased heart sounds were also observed.

Prior to the use of Doppler echocardiography in the hospital medical practice, carditis was revealed in 53 patients (81.5%) with ARF. After the Doppler echocardiography was introduced, carditis was diagnosed in 19 patients (95.0%): 12 of them (63.2%) had clinical manifestations, and 7 patients (36.8%) presented subclinical carditis.

Arthritis affected the large joints, mostly the knee joints (60.9%), rarely elbow, hip, ankle joints and, sometimes, shoulder, small joints of the wrist and foot. The majority of the patients with arthritis (37/ 80.4%) had migratory polyarthritis.

Sydenham’s chorea presented with hyperkinesis in all cases. Besides hyperkinesis, 18 children (81.8%) with chorea had hypotonia, 12 children (54.5%) had static and coordination disorders, and 11 (50.0%) suffered from psychological and emotional disturbances (behavior changes, emotional lability).

Erythema marginatum and rheumatic nodules were mostly observed in cases with high fever, intoxication, significantly increased inflammatory blood markers, and was usually associated with carditis.

Minor Jones criteria such as fever (65.9%), increased ESR (67.1%) and raised C-reactive protein (63.5%) occurred more frequently than arthralgia (p = 0.0008, p = 0.0006 and p = 0.0014, respectively). Prolonged PR interval on ECG was identified in 10 patients (11.8%).

All the patients had evidence of a preceding streptococcal infection. Raised anti-streptolysin O was found in 71.8% of the patients (n = 61); positive throat culture for Group A beta-hemolytic streptococci - in 27.6% (n = 8/ 29), recent streptococcal infection (tonsillopharyngitis, scarlet fever) was diagnosed in 55.3% of the children (n = 47).

The adequate treatment of preceding streptococcal infection was administered in 25 children (53.2%), mostly in children with tonsillitis and scarlet fever. It is likely that manifestations of pharyngitis were interpreted as those of viral etiology and antibiotic therapy was not administered.

A positive family history of ARF was determined in 12 patients (14.1%).

When diagnosed with ARF, the patients were receiving antimicrobial therapy for streptococcal infection. Penicillin was administered intramuscularly in 67 children (78.8%). Oral macrolides (erythromycin, clarithromycin, azithromycin) were administered in 15 children (17.6%). Other antibiotics were used in the remaining cases because of misdiagnosis on the admission to the hospital. The duration of antimicrobial therapy was of 10 days.

Nonsteroidal anti-inflammatory drugs were used in 78 ARF patients (91.8 %). The majority of them were treated with Diclofenac sodium (48/ 56.5%), while acetyl salicylic acid was rarely used (16/ 18.8%). Other nonsteroidal anti-inflammatory drugs (ibuprofen, nimesulide) were administered in the rest of the cases. Steroids were administered in 36 patients (42.4%), particularly in those with severe carditis. Anticonvulsant drugs (phenobarbital, carbamazepine) were given to patients with chorea.

Fatal cases were not reported among the ARF patients.

## Discussion

The mean age of the patients with ARF in our investigation corresponds to the most commonly reported age of the patients with streptococcal pharyngitis and is in agreement with the recently published findings [**3**]. In most populations, ARF and rheumatic heart disease are more common among females, presumably because they are more sensitive to streptococcus A, or potentially, due to the genetic predisposition [**11**].

During the study period, we have seen an average of 6.1 new cases of ARF per year, with significant outbreaks between 2000 and 2003 (11.75 cases per year). The number of ARF cases per year has been reduced 16.8 fold in the last 14 years (form 10.75 to 0.7), which correlates with the data on the overall ARF incidence in the Ternopil region. The publication from Montreal’s Pediatric Tertiary Care Centers reported an increase in the number of cases per year, from 1995 to 2005, as compared with the previous years [**8**].

The increased rate of admission of patients with ARF to hospitals from October to March, with the peak in March, is in agreement with the seasonal variability of pharyngitis, which is caused by group A beta-hemolytic streptococci and is most often diagnosed in the winter and spring [**11**] or in the fall and winter [**12**,**13**]. In contrast, pyoderma or skin infection occurs mostly in the summer and can be associated with acute glomerulonephritis [**14**]. The increased incidence of ARF in October may also be associated with the start of an academic year in September, which increases crowding and contact between schoolchildren.

Low temperature and wet weather can also be determining factors for the seasonality of ARF. The role of other factors, such as levels of hormones (especially of melatonin and glucocorticoids), self-reactive autoantibodies, inflammatory factors and the activity of the immune response could contribute to the seasonality of rheumatic diseases and is discussed in some publications [**15**].

Our data showed the frequency of rheumatic carditis of 84.7%, which is similar to the other published data, in which it ranges from 45% to 95% [**16**,**17**]. The use of Doppler echocardiography helps improve the detection of carditis. The Jones criteria for the diagnosis of ARF were revised in 2015 [**18**]. Echocardiography is now recommended in all the patients with suspected or confirmed ARF. Subclinical carditis can be used as a major criterion for ARF in all populations [**19**].

The reported frequency of arthritis in children with ARF ranges from 46 to 65%, depending on the region [**2**]. In our study, it was observed in 54.1% of the cases. Some authors [**3**] described arthritis as the most common criterion (up to 70% of the cases).

We diagnosed Sydenham’s chorea in 25.9% of the cases, although some authors reported the rates as low as 15-17% [**2**]. Some publications indicate rates of 6-31% and even 49% [**8**]. The difficulties in the ARF diagnosis were described in the cases of isolated rheumatic chorea, when other major criteria of ARF are absent [**6**].

The low rates of detected subcutaneous nodules and erythema marginatum in ARF children found in this study were likewise reported in literature [**2**,**3**].

The increased antibody titer to ASL-O ranges from 48.7% in Africa to 79.4% in the USA [**2**]. This is comparable with our results (71.8%). The positive throat culture for Group A beta-hemolytic streptococci in our study was present in 27.6%. It correlates with the data in literature, which indicate the frequency of positive culture in less than one third of the patients [**3**]. This can be explained by the existence of a latency period between pharyngitis and the onset of ARF. Other authors reported positive throat culture tests in 62.6% of the patients [**8**]. The interpretation of the data needed to confirm the streptococcal infection, frequently leads to the difficulties in establishing the diagnosis and making the differential diagnostics of ARF. Negative throat culture does not exclude the beta-hemolytic streptococci infection, which was confirmed in the literature [**10**,**11**]. At the same time, the positive throat culture can be present in the asymptomatic carriers of group A beta-hemolytic streptococci. The increased levels of antistreptococcal antibodies are not specific only for beta-hemolytic streptococci group A, but can also be seen during infections caused by other types of streptococci, which do not lead to ARF.

The high percentage of misdiagnoses at the time of admission indicates low level of ARF awareness by the family physicians and pediatricians, possibly because of the rarity of this disease. Atypical forms of the disease can also contribute to misdiagnosis.

In our opinion, the fact that only 53.2% of the patients received an adequate treatment of the preceding streptococcal infection deserves special attention. We recommend a more careful approach regarding the diagnosis of streptococcal tonsillopharyngitis, and the use of rapid antigen detection tests, which are currently not available in Ukraine.

Antimicrobial therapy for the treatment of ARF, which is currently used in Ukraine, is in accordance with international guidelines [**1**,**2**,**6**,**7**]. However, in Ukraine, as in other post-Soviet countries, a more accepted practice is to use particular nonsteroidal anti-inflammatory drugs such as Diclofenac sodium, rather than salicylates, in the treatment of ARF.

## Conclusions

The most frequent major Jones criteria of ARF in Western Ukraine are carditis (84.7%) and polyarthritis (54.1%). Chorea occurred significantly less frequently than carditis.

The significant incidence of misdiagnoses in ARF children during admission to the hospital, especially the interpretation of joint syndrome, indicates that physicians need extra awareness. The lack of specific clinical signs of rheumatic carditis makes it a diagnostic challenge. A revised version of the Jones criteria (2015) for the diagnosis of ARF can improve the detection of carditis.

The adequate treatment of the preceding streptococcal infection may prevent ARF.

**Sources of funding**

None. 

**Disclosures **

None. 
